# A qualitative evaluation of coproduction of research: ‘If you do it properly, you will get turbulence’

**DOI:** 10.1111/hex.13261

**Published:** 2021-05-05

**Authors:** Joanne Deborah Worsley, Mick McKeown, Timothy Wilson, Rhiannon Corcoran

**Affiliations:** ^1^ Department of Primary Care and Mental Health University of Liverpool Liverpool UK; ^2^ School of Nursing University of Central Lancashire Preston UK; ^3^ Applied Research Collaboration North West Coast (ARC NWC) Liverpool UK

**Keywords:** coproduction, involvement, lived experience, power, trust

## Abstract

**Background:**

Patients and public members are increasingly involved across the different stages of the research process. Their involvement is particularly important in the conception and design of applied health research where it enables people with lived experience to influence the aims, content, focus and methods.

**Objective:**

To evaluate the process of coproducing a mental health–related research proposal suitable for funding through a national health research funding body.

**Methods:**

Reflections from members of the public (n = 3) and academic researchers (n = 3) were collected through semi‐structured interviews. Data were thematically analysed.

**Results:**

Thematic analysis identified five overarching themes: valuing the lived experience perspective; matching ambitions to the funded research process; ‘Us and them’: power, relationships and trust; challenges; and benefits of coproduction.

**Conclusions:**

Our findings suggest that for successful coproduction of a research funding application, an open and trusting atmosphere, where equal relationships are established and a shared common goal agreed is essential. Although relationships with research professionals were framed by trust and mutual respect for some public advisors, others felt a sense of ‘us and them’. With various tensions played out through interpersonal conflict, difficult conversations and disagreements, coproduction was not a positive experience for all stakeholders involved. Among the learning was that when collaboration of this kind is constrained by time or funding, genuine, impactful coproduction can be more challenging than is generally acknowledged.

## 1 INTRODUCTION

Researchers increasingly endeavour to involve patients, carers and the public in their research. This process has become known as patient and public involvement (PPI). PPI, defined as research with and by patients, rather than to, for or about them, takes many forms; from identifying research priorities for funding, to interpreting and disseminating research findings.^1^ Active involvement of service users and members of the public in health research is now a central funding criterion for the National Institute for Health Research (NIHR). A growing body of evidence demonstrates benefits of PPI to research outcomes,^2^, ^3^, ^4^ ensuring that research ideas are genuinely useful for patients and carers.^5^ Patients and members of the public also accrue benefits including acquisition of new skills, personal development, support and friendship, and feelings of satisfaction and empowerment.^4^, ^6^, ^7^

Beresford^8^ identifies consumerist and democratic models of involvement in research. Consumerist efforts are ‘framed mainly in market research terms of ‘improving the product’’ (p.97), whereas democratic approaches are ‘primarily concerned with people having more say in agencies, organizations, and institutions which impact upon them and being able to exert more control over their own lives’ (p.97). Thus, consumerist approaches are generally ‘top‐down’, whereas democratic approaches are rooted in people's lived experiences and draw upon philosophies of human rights, participation, inclusion and autonomy.

A democratic impulse framed terms such as ‘user‐led’ or ‘user‐controlled’ research; however, the term ‘coproduction’ is increasingly used to describe a democratization formed around alliances between public and professionals.^9^, ^10^ Coproduction and service user involvement are also increasingly encouraged in mental health‐care research. For example, an evaluation of a service responsible for psychiatric care in Amsterdam involved a team of researchers and experts by experience collaborating in all phases of the study including the design, strategic decision making, data generation and interpretation. Although this enriched the conclusions and ideas for improvement, collaborative reflection on the participatory processes revealed complex relational dynamics in the coproduction of knowledge in such research.^11^

Coproduction is increasingly widely used in the design of applied health research, enabling people with lived experience to influence the aims, content, focus and methods. For example, Staniszewska and colleagues involved parents who had experience of having a pre‐term baby in the development of a research grant bid. As parents’ experiences and perspectives provided the focus for research meetings, user involvement impacted development of the research questions and methods. Good working relationships facilitated the process, whilst lack of financial support for public members, the time‐consuming nature of the process and professionalized research language were identified as barriers.^12^

According to Oliver and colleagues, however, coproduction is not free of cost or risk.^13^ Examples of practical costs associated with coproduction include managing group dynamics and administrative tasks such as arranging spaces.^13^ As many of the tensions are played out through interpersonal conflict, difficult conversations and/or disagreement, there are also significant personal costs to researchers.^13^ Many research professionals find navigating such tensions difficult, especially when research funding is at stake.

Despite a fundamental shift in expectations concerning public involvement in applied health research,^14^ examples of NIHR‐funded user‐led approaches to research are extremely rare.^15^ Researchers from the NIHR Collaboration for Leadership in Applied Health Research and Care North West Coast of England (CLAHRC NWC) sought to address this by enabling the development of a public‐led mental health–related research proposal suitable for funding through the NIHR Research for Patient Benefit (RfPB) programme. Funded by the Liverpool Clinical Commissioning Group Research Capability Fund (LCCG RCF), patients, carers and members of the public became co‐researchers and equal partners throughout the process of conceiving, agreeing, preparing and drafting an RfPB grant application including generating the ideas, design and applying for funding. This paper aims to evaluate this process. In particular, we explore people's appreciation of the process, motivations underpinning involvement, factors that may support or facilitate the process and challenges that hindered the process. Although previous research has evaluated experiences of coproduction in mental health‐care research (eg 11), this paper focuses on PPI in the very early stages of mental health research. Formalities of seeking research funding often constrain public voice in the process and can limit coproduction ideals. As PPI at early stages adds a layer of complexity, this paper provides guidance for those involved in coproduction processes.

## 2 METHODS

### 2.1 Process

The NIHR CLAHRC in the North West Coast (NWC) of England funded a series of three public engagement events called ‘Research: Have your say’, which were held during 2015/2016. The aim of the events was to initiate research into inequalities in mental health care led by service users and the public across the NWC region. These events involved a mix of presentations, group work and open discussions. In line with the James Lind Alliance, service users and members of the public were asked to identify and prioritize their research ideas. These substantially coalesced around increasing understanding between mental health service users and mental health‐care professionals to reduce stigma, and improving the quality and appropriateness of the care provided. From an initial 80 participants involved in scoping priorities, smaller groups of between 20 and 30 participants met to refine ideas, until 11 agreed to form a working group to develop the research proposal. Throughout the process, public advisors’ (the generic term utilized within the CLAHRC to refer to people involved in PPI) experiences and perspectives provided the focus for the working group meetings. Although the broad research area was settled upon during the ‘Research: Have your say’ events, four full‐day workshops utilizing participatory and democratic facilitation methods were held in 2016/2017 to assist the working group to align their research affinities with the priorities and criteria of a relevant national funding body, distilling ideas towards a workable research project. This systematic process of service user and carer engagement identified the psychiatric encounter as the key focus for the research proposal. The aim of the eventually submitted research proposal was to improve the quality of therapeutic relationships between psychiatrist and service user within routine clinical encounters.

### 2.2 Participants

The working group initially comprised four research professionals and 11 service users, carers and members of the public. Only five public advisors sustained involvement throughout the entire process, becoming co‐applicants on the research proposal. Research professionals and those public advisors who sustained involvement were invited to participate via email. Reflections from three applied health research professionals (2 men, 1 woman) and three public advisors (2 men, 1 woman) were gathered using semi‐structured interviews. Our public advisor sample comprised individuals self‐identifying as service user, service user/carer and member of the public. Some members of the academic team combined a background in academic research with lived experiences as service users and/or formal or informal carers. All participants, to varying degrees, take a critical view of contemporary psychiatric services, particularly around issues of compulsion and coercion. Data collection was carried out in June 2020 following ethical approval to proceed with the study from the University's Health and Life Sciences Research Ethics Committee (5582). Informed consent was obtained from all participants.

### 2.3 Interview

The semi‐structured interview was co‐designed by the first author with the public advisor author. The schedule comprised four sections focusing on motivations for being involved; the agreed research proposal; appreciation of the process; and reaction to the received feedback from the funding organization (see Supplementary file S1).

### 2.4 Analysis

Data were collected and thematically analysed by a researcher independent of the bid development process, with no prior relationships with the public advisors. Thematic analysis was chosen as it is a qualitative method that aims to identify, analyse and report distinct or recurrent themes in data.^16^ This analysis takes a realist epistemological standpoint, treating participants’ narratives as representative of their lived reality. Line‐by‐line coding derived from a largely inductive approach ensured that data were not overlooked. Although line‐by‐line coding was undertaken by the first author, the first and fourth author met throughout the coding process to examine emerging impressions of the data. The initial themes captured by coding were refined during discussions to produce the final themes. This process ensured the final themes were not just the personal interpretation of one team member.

## 3 RESULTS

Our analysis offers five broad themes, titled: valuing the lived experience perspective; matching ambitions to the funded research process; ‘Us and them’: power, relationships and trust; challenges; and benefits of coproduction. The research professionals’ quotes are denoted by ‘R’ and the public advisors by ‘PA’.

### 3.1 Valuing the lived experience perspective

Public advisors’ reflections around motivation were dominated by efforts to change the mental health system, not just for oneself but also for others:

Let’s do this. Let’s do it for Liverpool. Let’s do it for everybody, the whole nation. We’ve come up with something for improving mental health. It’s that sort of attitude (PA2).

Most participants had misgivings about standard psychiatry, with some recounting negative experiences of psychiatric consultations, which underpinned their passion to see change enacted in the mental health system:

They [referring to psychiatrists] can make you angry. I got up to show the psychiatrist how my writing had changed and he said ‘sit down sit down’ as if he was frightened I was getting too close to him (PA2).

Public advisors were therefore able to articulate a true essence of experience, which is not available from other sources. In relation to providing experiential expertise, payment for involvement work was highlighted:

I think payment should be given if they’re wanting your expertise and calling you an ‘expert by experience’. I don’t want to be an expert in some of the experiences I’ve had’ (PA3).

‘People are doing pieces of work, they are acting like researchers, they are using their experience in the same way as paid researchers use their experience, their expertise or their knowledge (PA2).

### 3.2 Matching ambitions to the funded research process

Some public advisors advocated that certain care and treatment approaches should be emphasized. However, the professional researchers involved in the process were mindful of the type of research that would be suitable for NIHR funding:

At least one of the people was a real zealot for a form of family intervention called Open Dialogue. So they were really wedded to the idea that the group should be doing a project around Open Dialogue … In actual fact, for me personally, I thought it was a great idea and I’m really keen on those approaches but I was also aware the Research for Patient Benefit were already funding a trial on Open Dialogue in the UK so they were unlikely to fund us to do it (R2).

Although the research system supports and encourages coproduced research with patients and members of the public, constraints exist that preclude focusing on issues that public advisors may find most pressing. For example, one public advisor was on a quest to transform psychiatry:

With me it’s got to be an alternative to psychiatry because that is my standpoint. I know how dangerous and the bad things it does to people so I’ve got a distinct standpoint. I tend to think will this action lead to an abolition of psychiatry? (PA1).

Through the process, it became apparent that some issues public advisors were passionate about would not be suitable for NIHR funding:

There is a big tension between what people might want and what might actually be fundable. There was a perpetual collision between expert advice and lay expectations … legitimately people get involved in this sort of stuff because they want to shake things up, they want to change things and then research may be the poor relation of social change (R2).

A few times I was asked to speak in that capacity to say actually this is what the remit for the RfPB is, this is NIHR’s viewpoint therefore this is the constraint that you’re working in. For me that created some of the tricky bits because people were activists, they wanted the system to change completely (R1).

The navigation and negotiation towards convergence between meeting the desires of the public advisors and the understood remit of the funding organization proceeded in small but significant steps. For example, although his idea was not taken up because it was felt to be too radical, one participant made the decision to remain in the working group with the hope that the research might engender small changes if selected for funding:

It [referring to psychiatric consultations as the selected focus for inquiry] was one of the last things I would have gone for but when they decided 11‐1, I fully agreed because I’m very passionate about mental health so I fully went with them… I worked with them and supported them, thinking in the long term that these consultations may change (PA1).

Although the direction of the research proposal was a compromise and did not reflect the main priorities of all stakeholders, public advisors demonstrated a capacity to compromise and co‐operate throughout the process. Thus, self‐development as a collaborative applied researcher was evident.

### 3.3 ‘Us and them’: power, relationships and trust

Good working relationships between academic researchers and public advisors were identified as a key enabler. These were characterized by communication, support for each other and the aim of the proposal, and the perceived absence of a power hierarchy. This perception of equality was crucial in developing openness, and public advisors were encouraged to express their views and opinions throughout the process:

In terms of the other members of the team, particularly the academics, they extremely allowed us to express our points of view and it was open (PA1).

I felt as though we were on an equal base at those meetings (PA2).

Importantly, public advisors felt as though the research professionals took time to listen to their narratives and respected their points of view. Research professionals therefore helped public advisors feel comfortable. A safe space was established as trust and mutual respect grew over time:

Since I’ve been in with this research and the people, I’ve felt as though I can open up and I could talk. They know more about me and that’s given me trust to be able to talk (PA3).

I think the mutual respect was very important so I respected their point of view and they respected my point of view (R1).

However, due to budgetary constraints, public advisors were not involved in the administrative aspects of the process, which may have translated into an imbalance of power:

There were a set of us who looked like decision makers around where the meetings will take place, how often will they happen, what’s the content of those meetings, what we are going to cover so that didn’t probably feel as democratic as it could have done… I think that that, in turn, translated into an imbalance of power (R3).

As researchers were also cognisant of time constraints throughout the process, they felt pressured to progress the discussion:

We always ended up going over the same ground so it sometimes felt like we spent half the session talking about the issues we had already covered so it was quite difficult to move it forward. Sometimes if I was feeling the frustration, I would feel as though I had to get bossy and try to move it forward (R3).

However, attempts to progress the discussion may have been perceived as researchers closing down conversations on particular topics. Indeed, some public advisors thought that the researchers were trying to steer the meetings in a certain direction:

There were some who were just a bit nasty who thought the academics had their own agenda (PA1).

There was one lady that was really dominant and was better educated than myself but she thought all of the academics were taking over (PA2).

As some public members felt their views were marginalized, this created for some a sense of ‘us and them’:

What was stressful was that some of the public advisors, not the academics, the other members of the group were very critical of some of the academics and I didn’t agree with their point of view because I thought everything was very open (PA1).

There was an imbalance for members of the public because academic voices can be very loud so that was an issue throughout it. But probably the biggest issue was to do with trust… We didn’t have those conversations about who we are so it almost became about a bunch of academics and a bunch of public advisors and we are trying to put this piece of research together rather than there’s a bunch of people here who are really interested in improving mental health, they each have different sets of experience and expertise that they would like to bring to bear on this. We didn’t have those initial discussions that would have established a better sense of trust, and for that reason this idea of an ‘us and them’ began to emerge (R3).

Beginning the process with ‘healing’ sessions to deal with the perceived or real power divide would have allowed for the building of greater trust and understanding to avoid negative dynamics:

It was only in an ad hoc meeting that we called to try and address this and we sat round literally in a circle and we unloaded and all sorts of things were picked up on pretty much everyone’s experience around the circle of having either carer or lived experience of mental distress. It was that process that really enabled us to take this process forward I think… If I was to do it again I’d make sure that I built in enough time for that trust and that knowledge of the group, that group‐ness, the we‐ness is established before you try and do anything as complicated as designing a piece of research asking a really political and complex question (R3).

To some extent if you’re really going to get to user‐led research that’s in alliance with people who have previously used services and implement this stuff in a way that changes services, we’ve got to do, and I don’t want this to sound too psychobabble, but a sort of healing process between each other. We want to get over all the upsets that people have got because they are the things that have driven people into this territory in the first place. I think we can’t have as good a level of trust as we might if we haven’t done that sort of truth telling (R2).

Given people's grievances, healing sessions are also important to deal with the ‘hurts’ dealt to some by the system.

### 3.4 Challenges

#### 3.4.1 Barriers to user involvement

While some factors, such as good working relationships, facilitated the process, there were also a number of challenges. One challenge was the professionalized language of research. Despite being alert to the hazard, academic researchers could lapse at times:

Although we thought that we were pretty good at communicating ideas without using academic type language … You’re never aware that you slip into it subconsciously all the time because it’s a professionalised language (R3).

In line with this, some public advisors struggled to understand certain aspects of the process. In particular, public advisors grappled with the some of the chosen methods; despite a consensus for a Delphi approach designed to equally respect lay and professional expertise:

The process of the Delphi, I couldn’t get my head around it (PA2).

#### 3.4.2 Challenges specific to the group

Working collaboratively towards a well‐defined goal with people whose life‐experience has been characterized by interpersonal trauma and/or structural troubles is challenging:

Mental distress affects people in ways that affects how they relate to other people… I think one of the key principles of a deliberative democracy is to try and take your own personal feelings out of disagreement and it’s easier to do that if you’re stable yourself and steeped in a psychosocial understanding of relationships. If you’ve had a horrible, awful life that might have involved being abused as a child and your everyday makeup is to mistrust people and see threats in disagreements and your usual response is to bite back or get your equaliser in first and to do everything you can that breaks a relationship but at least the breaking of a relationship you’re in charge of it (R2).

If you are working with an unhappy bunch of service users you are going to get stick from them… Sometimes they can come out with things that are quite hurtful (PA3).

Some public advisors struggled to retain knowledge from one session to the next. As a consequence, repetition was common throughout the process:

There’s a mismatch between an expectation that you can take a topic and deal with it within a session and then move onto another session and deal with that in a session and not realise that in between those meetings, in between that was all lost because other things were happening like ‘I can’t pay my rent’ or any other number of life things were coming to interfere with the process as well as feeling in distress as well as potentially having symptoms (R3).

#### 3.4.3 Structural impediments to meaningful PPI

Although patient and public involvement is encouraged by many national funding bodies, research professionals are still expected to write a grant proposal reflecting the research paradigm. Adhering to NIHR requirements, a research‐orientated proposal, rather than a user‐friendly text suitable for patient and public members, was produced:

When it’s coming from the professionals, ok the professionals are writing what the service users have said, but sometimes it’s done that much in their own words that the service users don’t understand it (PA3).

Although there has been a shift in expectations about public involvement in applied health research, the feeling that the NIHR is yet to demonstrate full commitment to coproduction was articulated:

Despite all the rhetoric around NIHR for public engagement, and you get to write about this on one of the boxes on the forms, I think the system’s commitment to an authentic messy public engagement is not there yet. If your public engagement gets you to a pristine application that looks like it was written by a bunch of academics, that’s what they want but ours didn’t turn out like that (R2).

A separate funding stream for authentic coproduction was felt to be needed, enabling the adoption of methods that do not align with positivist research values:

There should be a different funding pot that should be for scruffy user alliance projects that may not be pristine science (R2).

Implicit to this process was that there had to be a singular output, which was a grant proposal reflecting the research paradigm. This underpinning output, destination and constraint was problematic because, although the likelihood of rejection was talked about often in the group, public advisors were disheartened by the feedback from the NIHR:

That was disappointing. There was a hell of a lot of effort. I actually wrote about a 100‐page proposal on my ideas (PA1).

At what scale do you put in effort that’s been put into it. All the thinking, again it was coproduced, but the academic side of it and all the help and the work that they put into it. I thought it was very disappointing (PA2).

Although there were layers of constraint on the discussion, including meeting the requirements of a formal grant proposal, time constraints were also felt, perhaps as a consequence of using a seedcorn grant from the LCCG to fund the process:

It just limits the amount of time you’ve got to be able to do something like this. It becomes a pressured thing rather than a wanted thing and a necessary thing (R3).

As true coproduction takes time, the process felt rushed towards the end. This was due, in part, to the way in which public advisors were funded:

It didn't end up being as good as it could have been because we just simply didn't have the time to make it so … Time constraints and if you like the capacity of our public advisors to maintain that track towards because of the way that they were funded to enable this process (R3).

Paradoxically, if you try and do the right thing by people in terms of paying them and you’ve only got a limited budget, the amount of times you can come together is limited (R2).

Mechanisms to fund similar processes need to be carefully considered as it may be more appropriate to employ patients, carers and members of the public as lay researchers in a more substantive way:

If you want to really meaningfully involve people with lived experience with mental health or any other experience actually you have to employ them (R3).

### 3.5 Benefits of coproduction

Despite the very real and diverse challenges, involvement practices were recounted as having a positive impact. Public advisors enjoyed the process and felt energized after the research meetings:

I could talk at these meetings and when I came back I was bouncing. I had energy (PA3).

Social aspects associated with membership of the working group were highlighted by both researchers and public advisors:

I think we developed good camaraderie’ (PA1).

‘Some of the solidarity and positive connections that were built in that process were unquestionably good things (R2).

As the process provided a setting where public advisors exchanged experiences with peers, this enabled them to feel comfortable with their own experiences and thought processes, and some felt less alone as a consequence of listening to other people's narratives:

The identification of others… When they shared their experiences I can think I’m not the only one who thinks like that (PA3).

Last, the involvement of patient and public members had an important impact on the development of the research aim, and a grant application was coproduced rooted in people's experiences, whilst also addressing key research questions selected by the group:

We wouldn’t have hit upon the topic we did without the frank discussions of what it’s like to be in a psychiatric consultation … we certainly wouldn’t have hit upon that as the real target for our research bid… We had a proper feel of the question we were asking. You don’t often have that unless you have coproduction with proper embedded lived experience (R3).

## 4 DISCUSSION

The aim of this paper was to evaluate the process of coproducing a mental health research proposal suitable for funding through a national health research funding body. Efforts were made to coproduce this research proposal in its entirety and to address power imbalances between the applied health research professionals and public advisors. Our findings suggest that for successful coproduction of a research funding application, an open and trusting atmosphere, where equal relationships are established with shared common goals are essential. Different forms of expertise were seen to be equally valid, and a plurality of views and opinions were expressed throughout the process. In light of this, some public advisors felt as though they were treated as equals, which contributed to an atmosphere of mutual respect and trust. Although this was true for some, others felt a sense of ‘us and them’. With various tensions borne out through interpersonal conflict, difficult conversations or disagreements, participants, including the research professionals, often struggled to manage relationships. Although the research system encourages coproduction, our findings suggest constraints exist in that system that preclude focusing on the issues that service users and members of the public find most pressing. Our findings further suggest that even within a small working group, there are competing priorities. Although the direction of the research proposal did not reflect the main priorities of all stakeholders, a capacity to compromise and co‐operate was evident during the process. We found that when collaboration of this kind is constrained by time or funding, genuine, impactful coproduction can be more challenging than is generally acknowledged.

Certain recommendations flow from the findings. Beginning the process with ‘healing sessions’ where trusted relationships are afforded time to develop may reduce negative impacts and enable the process to deliver more benefits than costs.^17^ As tensions come in and out, ‘healing sessions’ could also be scheduled responsively throughout the process. For researchers and public advisors to work together as equals within a research team, public advisors could be employed as lay researchers. This may further reduce divisions and can deepen mutual understanding and recognition, including matters of dual identity.^18^

Constraints on PPI within funded projects are well known, wherein participatory ideals can clash with tight regulation, obligations to external stakeholders and inflexible deadlines.^19^ Critical inquiry has drawn attention to processes of legitimation and de‐legitimation whereby divergent views from accepted orthodoxies can be neutralized within powerfully present cultures, such as those which obtain in everyday practices of research.^20^, ^21^ Our findings, relating to PPI seeking funding, confirm such observations and suggest that using a collaborative process for a singular, well‐defined and inflexible output should be avoided. Instead, researchers should engage with a plural set of outcomes so that failure of one outcome can be countered by success of others. It would also be beneficial to plan a strategy, which could include a number of different sources of possible funding so that the team could view submission processes as opportunities to receive formative feedback in order to avoid feelings of disappointment.^12^ In line with this, funding bodies could adopt a more flexible perspective by considering how grant applications could be written in a more user‐friendly way and reflecting on how the process of feedback can be handled to deliver the opportunity to build capacity in public, patient or carer researchers.^12^ Separate funding streams may better serve public involvement in research to be founded on authentic coproduction instead of partial inclusion or tokenistic engagement.

The reported research has limitations. First, although eleven service users and members of the public from across the NWC of England agreed to form a working group to develop the research proposal, only three participated in this evaluation. Thus, the findings are limited to those service users and members of the public who were possibly more motivated and may not be representative of the views held by all participants. Similarly, as only three applied health research professionals participated in the evaluation, the views from these individuals may not be representative of the experiences of all research professionals involved. That said, the six participants did constitute a reasonable fraction of the total number of participants in the project.

In sum, the process used to develop the research proposal has highlighted the complexity of involving patients, carers and members of the public in the conception, design and development of applied mental health research. Some public advisors and professional researchers derived clear benefits from the process, whereas others would have benefited from a less structured more flexible and open process where time allowed for the building of greater trust and understanding to avoid an ‘us and them’ dynamic. There were undoubtedly some challenges to ideals for a relational democracy within the group. Yet, despite some turbulence along the way not all of this was negative, and some tensions and tribulations may even indicate a degree of success in creating a space for free and honest communication, with pointed or heated contributions not to be discounted.^22^ There remains, however, a need to bring together diverse and contentious perspectives under a process of careful deliberation that facilitates ease, confidence and concern for each other among all present.^23^, ^24^

Beginning the process with a reparative session to deal with perceived or real power differentials as well as with the ‘hurts’ experienced within mental health care and research may have minimized negative impacts and enabled the process to maximize benefits. To conclude, establishing trust from the outset enables different perspectives and opinions to be expressed and challenged, which subsequently can thoroughly enrich the research process and its outputs.

## 5 Patient and public contribution

A public advisor was involved in the planning stages and designing the interview topic guide.

## 6 ACKNOWLEDGEMENTS

This research is supported by the National Institute for Health Research Applied Research Collaboration North West Coast (NIHR ARC NWC). The views expressed in this publication are those of the author(s) and not necessarily those of the National Institute for Health Research or the Department of Health and Social Care.

## CONFLICT OF INTEREST

None.

## Supporting information

Supplementary file S1

## Data Availability

Qualitative data extracts are presented in the article to support the findings. The original transcripts are not available to the public as they may contain information that could compromise the confidentiality and anonymity of the participants.
