# Supplementary material for: A qualitative evaluation of coproduction of research: ‘If you do it properly, you will get turbulence’
Source: Health Expect. 2021 May 5;25(5):2034–42. doi: 10.1111/hex.13261 (PMC9615072; doi:10.1111/hex.13261)
Supplement: Supplementary file 1 — Supplementary file S1 [file HEX-25--s001.docx]

**Interview schedule**

**Section 1: People’s route in and personal motivations**

- Do you remember the series of public engagement events? These engagement events were called ‘Research: Have Your Say’.
  - Can you tell me what these events were about?
  - How did you get involved?
  - Why did you get involved? / Why did you go to these events?
- Do you remember when you used to meet as a working group?
  - What are your recollections about what the group was trying to do?
  - What did you think the point of it was?

**Section 2: Sense that people made of the research proposal**

- The group were trying to construct a grant proposal. Can you tell me what the proposal was about?
- Did you think that the proposal would be selected for funding?
  - If yes, why?
  - If no, why not?
- What do you think the proposal should have been about? What subject, issue or area?
  - What was this like for you when your idea was not taken up within the democracy of the group?
  - Can you recall why the group did not go with your idea?
- Would you have done anything different?
  - Can you tell me more about that? / If so, what would you have done differently?
- What did it feel like being a member of the public in the room?
- Everyone had different points of view, what did you think about the debate that happened?
  - Why do you think people behaved like that?

**Section 3: People’s appreciation of the process**

- How did you feel about the process of putting together a proposal?
  - Why did you feel that way?
- Did you feel involved in the whole process?
  - If so, what made you feel involved?
  - If not, what made you feel disconnected from the process?
- What did you bring to that process?
  - Did you feel as though you had an input/a say about the direction?
- What did you like about the process?
- What did you not like about the process?
- What did you gain from the process?
  - Did you learn anything? If so, can you tell me about that?
- Would you do something like this again? Why? / Why not?
- Would you feel prepared to do something like this again?

**Section 4: Feedback and impact**

- How do you feel about the proposal not being funded?
- Here is a copy of the feedback. Is this a fair criticism of what the group did?
- What has happened since you participated in this process? / Has this process had any impact on what you are doing now?
- Do you have any additional comments you would like to make about the research process?
